# *CHEK2* mutations and papillary thyroid cancer: correlation or coincidence?

**DOI:** 10.1186/s13053-022-00211-7

**Published:** 2022-01-31

**Authors:** Kortbeek Koen, De Putter Robin, Naert Eline

**Affiliations:** 1 Department of Medical Oncology, University Hospital Ghent, Ghent, Belgium; 2 Department of Medical Genetics, University Hospital Ghent, Ghent, Belgium

**Keywords:** *CHEK2*, *CHEK2* c.1100delC, Papillary thyroid Cancer, Breast Cancer, Thyroid Cancer screening

## Abstract

We report the case of a breast cancer survivor, diagnosed with an underlying *CHEK2* c.1100delC heterozygosity, who developed a papillary thyroid cancer 5 years later. A *CHEK2* c.1100delC (likely) pathogenic variant is associated with an increased risk of breast, prostate and colorectal cancer and therefore risk-specific screening will be offered. Current national and international screening guidelines do not recommend routine screening for thyroid cancer. Hence, we reviewed the literature to explore the possible association between a *CHEK2* mutation and thyroid cancer. A weak association was found between the various *CHEK2* mutations and papillary thyroid cancer. The evidence for an association with *CHEK2* c.1100delC in particular is the least robust. In conclusion, there is insufficient evidence to warrant systematic thyroid screening in CHEK2 carriers.

## Introduction

### Case presentation

In 2014, our patient was diagnosed with cancer of the left breast at the age of 35. She was treated with a mastectomy accompanied by an axillary lymph node dissection, following a positive sentinel node biopsy. Definitive pathological staging showed a grade 3 invasive ductal carcinoma no special type pT1c pN1a (TNM 7th Edition). Additional pathological characteristics revealed a ki67 of 10%, a positive hormone receptor status and no *HER2Neu* amplification. She received adjuvant chemotherapy, radiotherapy and 5 years of tamoxifen in combination with a LHRH agonist.

**Fig. 1:**
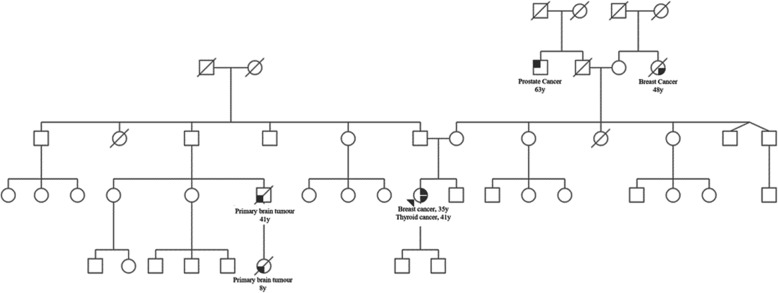
Case pedigree. Proband indicated with arrow

As recommended by national guidelines, genetic screening was performed because of her young age which found a germline heterozygosity for *CHEK2* c.1100delC. The gene panel included *BRCA1*, *BRCA2*, *PALB2* and *CHEK2* c.1100delC. Maternal familial history revealed a female with breast cancer at age 48 and a male with prostate cancer at age 64, both are third degree relatives. In the paternal family history, there were two cases of a primary brain tumour in a second and third degree male relative. The *CHEK2* variant was found to be paternally inherited. Her two sisters were tested and did not share the mutation, further family members have not been tested. Considering the increased risk of contralateral breast cancer associated with this germline *CHEK2* mutation, the patient opted for a preventive contralateral mastectomy with bilateral breast reconstruction. (Fig. 1)

In March 2020 clinical examination revealed a cervical adenopathy and a Fluorodeoxyglucose (FDG) Positron Emission Tomography – Computed Tomography (PET-CT) was performed, which showed a necrotic adenopathy in region 2A right and a heterogeneous, metabolically active lymph node in region 3 right. Excision biopsy of the lesion in region 2A revealed a lymph node metastasis of a papillary thyroid carcinoma. A total thyroidectomy with cervical lymph node dissection of the right regions 2–5 was performed. The pathology report confirmed the presence of an invasive papillary thyroid carcinoma (pT3N1b (TNM 8th Edition)). In June 2020 an adjuvant treatment with 100 mCi I^131^ was administered with curative intent. Until now, there is no evidence of relapse.

This case led us to review the evidence for an association between mutations in *CHEK2* and papillary thyroid cancer. In particular, does the *CHEK2* c.1100delC mutation convey a higher risk of papillary thyroid cancer and how should we counsel these women?

## Background

**Fig. 2:**
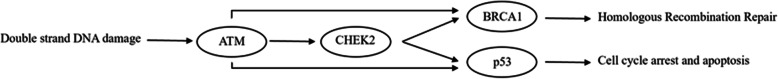
Simplified ATM-CHEK2-BRCA1 pathway. In the presence of double strand DNA breaks, sensor protein complexes activate ATM. ATM leads to phosphorylation of CHEK2 and p53 stabilization. CHEK2 also phosphorylates p53 and several other proteins contributing to p53 dependent cell cycle arrest, which lead to apoptosis. CHEK2 activates BRCA1 and other regulators by phosphorylation, leading to the formation of homologous recombination repair complexes. Image courtesy of author

*CHEK2* is a gene located on chromosome 22q and acts as a tumour suppressor gene. It encodes for the protein CHEK2, the human ortholog of yeast Cds1 and Rad 53, which are G2 checkpoint kinases. DNA double strand breaks lead to activation of ATM kinase, which in turn activates CHEK2 by phosphorylation of the N-terminal regulatory domain. CHEK2 phosphorylates p53, mediating activation and stabilization of p53 by ATM. CHEK 2 also phosphorylates BRCA1, modulating its function towards homologous recombination DNA repair, as well as several other regulators. As such *CHEK2* is a member of the homologous recombination genes involved in the DNA repair pathway [1–3]. (Fig. 2)

**Fig. 3:**
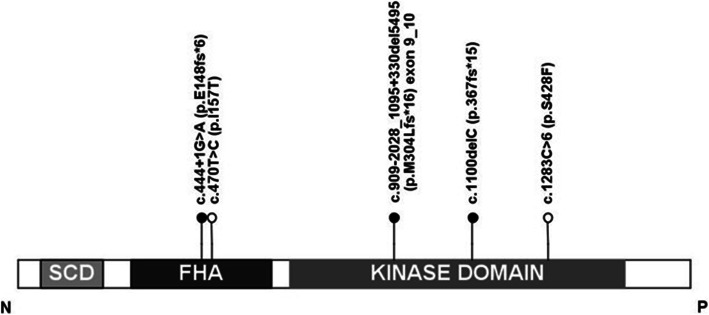
CHEK 2 protein domains. Lollipops indicate sites of founder mutations (black: truncating; white: missense). (SCD: SQ/TQ cluster domain; FHA: forkhead-associated domain; KD: kinase domain). Image courtesy of author

The majority of the studies evaluating the risk of cancer conferred by *CHEK2* mutations have focused on two *CHEK2* variants: c.1100delC and c.470 T > C (p.Ile157Thr, hereafter referred to as I157T), which are most prevalent in the European population. Other founder mutations exist and include c.444 + 1G > A (IVS2 + 1G > A), deletion of exons 9–10 (also known as EX8_9del and del5395) and the Ashkenazi Jewish founder variant c.1283C > T (p.S428F). Throughout the gene pathogenic truncating, missense, and splicing variants have been documented [3, 4]. (Fig. 3)

*CHEK2* c.1100delC is a (likely) pathogenic variant of the *CHEK2* gene caused by deletion of a single cytosine. This results in truncation, and loss of CHEK2 activity [5]. Heterozygosity for this mutation is found in 0,5 – 1,4% of the Northern European population [5, 6]. Case-control studies found an association with breast, prostate and colorectal cancer [5]. A large meta-analysis found a 3 to 5 times higher risk of breast cancer [5]. Other truncating *CHEK2* mutations showed a similar risk [7].

The missense I157T mutation is mainly found in Central European populations with a carrier frequency of 5–6% [3, 7]. One study found an increased risk of breast, colon, kidney and prostate cancer associated with this variant [7].

## Materials and methods

**Fig. 4:**
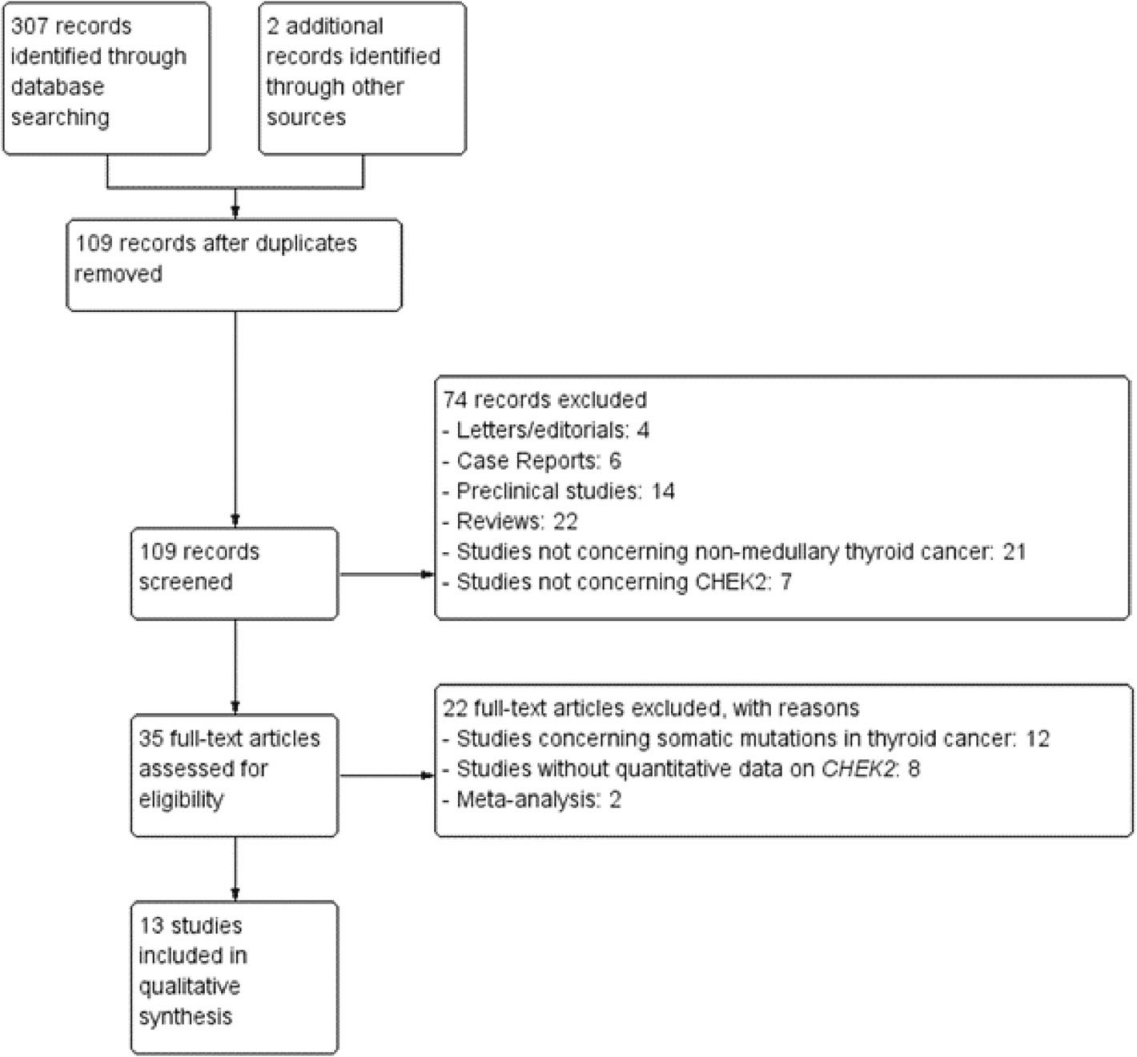
Search Strategy Diagram

We performed a comprehensive search using multiple databases (MEDLINE, Embase, Google Scholar, and Web of Science) until 15 February 2021. The search was restricted to studies in English, French, Dutch and German. Search terms were “CHEK2”, “CHK2”, “rad53”, and “Thyroid Cancer”. Search strategy diagram is provided. Reviews, meta-analyses, case reports, preclinical data and letters were excluded. Studies concerning medullary thyroid cancer were excluded considering the separate genetical background of this pathology. Studies reporting only somatic mutations in thyroid cancer were excluded. One study was rejected because the same dataset was previously included [8, 9]. (Fig. 3 and 4)

## Results

The evidence of a possible association between *CHEK2* mutations and papillary thyroid cancer is derived from 5 different types of studies:

### Incidence of papillary thyroid cancer in CHEK2 c.1100delC mutation carriers versus non-carriers

The Copenhagen retrospective cohort study reported the incidence of different types of cancer in a cohort of 670 carriers of a heterozygous *CHEK2* c.1100delC mutation in comparison to a cohort of 86.305 non-carriers. Cancer diagnoses were retrieved by linking the individuals to the Danish Cancer Registry. The median follow-up time was 43 years. The incidence of thyroid cancer was slightly higher in the group of *CHEK2* c.1100delC mutation carriers (5/670 (0,9%) vs 99/86303 (0,1%)). The age and sex adjusted hazard ratio (HR) however was not significantly increased (HR 1,26; 95% CI [0,18 – 9,09]; *p* = 0,81) [10].

A large cross-sectional study evaluated the frequency of different cancer types in germline carriers of (likely) pathogenic *CHEK2* variants versus non-carriers. These carriers were identified by multigene panel testing. The reason for multigene panel testing was not disclosed. Frequency of cancer was extracted from the patient history. The population was predominantly female (90%), with mainly Caucasian (75%) and Ashkenazi Jew (10%) ethnicity. This study identified 1101 patients with a *CHEK2* (likely) pathogenic variant, 76% founder and 24% non-founder variants. The most frequent founder mutations were c.1100delC (49%), I157T (30%), S428F (12%), Ex8_9del (3,5%) and c.444 + 1G > A (3,5%). The frequency of thyroid cancer in *CHEK2* mutation carriers (excluding I157T) was 3,7% compared to 2,1% in the group of 31,080 non-carriers, which implicates a significantly higher odds ratio (OR) for developing thyroid cancer when a *CHEK2* mutation is present (OR 1,77; 95% CI [1,15 – 2,62]; *p* = 0,01). There was no difference in frequency between founder and non-founder mutations. Thyroid cancer histology was not provided [11].

### Prevalence of thyroid cancer in CHEK2 c.1100delC mutation carriers versus other CHEK2 mutations

A recent retrospective study in patients who underwent hereditary cancer multigene panel testing identified 2508 carriers of a pathogenic *CHEK2* variant. There were 119 unique *CHEK2* variants identified, whereof the most prevalent variants were c.1100delC (31%), p.I157T (27%), c.1283C > T, deletion exons 9–10 (3,4%), c.444 + 1G > A (3%), c.1427C > T (5%), c.349A > G (4,6%) and c.190G > A (3,2%). The population was mainly Caucasian (80%), with about 9% Ashkenazi Jew ancestry. The frequency of thyroid cancer (histology not specified) was 2,9% in all variants combined and was not significantly different compared to c.1100delC. The highest frequency of thyroid cancer was reported in c.444 + 1G > A (5,8%) and c.190G > A (5,6%) carriers [4]. This study lacked a non-carrier control group.

#### Prevalence of thyroid cancer in germline CHEK 2 carrier case series

One small study examined the use of ultrasound screening for thyroid cancer in 62 female carriers of *CHEK2* truncating mutations (c.1100delC, IVS2 + 1G > A, del5395). Nodular goiter was diagnosed in 60%, fine needle aspiration was performed in 40% of all patients and papillary thyroid cancer was subsequently found in 6% (4/62). There was no control group [12]. One conference abstract reported 107 *CHEK2* mutation carriers identified through next generation sequencing in a North American Caucasian, mainly female population. The majority of these patients had a history of cancer (59%), mainly breast cancer. A history of papillary thyroid cancer was reported in 4%. The specific *CHEK2* variants in thyroid cancer cases were not reported [13].

### Prevalence of germline CHEK2 mutations in thyroid cancer patients versus healthy matched controls

Six studies compared the prevalence of germline *CHEK2* mutations in patients with thyroid cancer with a matched cohort (Table 1).

**Table 1:** Prevalence of *CHEK2* mutation in patients with thyroid cancer versus healthy controls based on case-control series. (NA: no data available; OR: odds ratio; CI: confidence interval)

Author	Population	Tumor Type	Investigated Gene Variant	Carrier Frequency in thyroid cancer patients		Carrier Frequency in healthy controls		OR	95%-CI	*p*-value
Cybulski et al. (7)	Polish	Papillary thyroid	c.1100delC	1/173	(0,57%)	10/4000	(0,25%)	2,3	[NA]	0,9
			c.444 + 1G > A	5/173	(2,89%)	19/4000	(0,48%)	6,2	[NA]	0,0003
			c.470 T > C	15/173	(8,67%)	192/4000	(4,80%)	1,9	[NA]	0,04
Siołek et al. (8)	Polish	Papillary thyroid	c.1100delC	1/468	(0,21%)	0/468	(0,00%)	NA	[NA]	NA
			c.444 + 1G > A	10/468	(2,14%)	1/468	(0,20%)	10	[1,3 – 78,1]	0,03
			c.470 T > C	60/468	(12,82%)	25/468	(5,30%)	2,8	[0,6 – 14,8]	0,001
			c.27417113-27422508del	6/468	(1,28%)	2/468	(0,40%)	3.0	[1,7-4, 6]	0,2
Fayaz et al. (15)	Iranian	Non anaplastic thyroid	c.444 + 1G > A	0/100	(0%)	0/ 100	(0,00%)	NA	[NA]	NA
			c.470 T > C	0/100	(0%)	0/ 100	(0,00%)	NA	[NA]	NA
Wojcicka et al. (16)	Polish	Papillary thyroid	c.470 T > C	169/1700	(9,94%)	98/2056	(4,70%)	2,2	[1,71 – 2,86]	< 0,0001
Kaczmarek-Rys et al. (17)	Polish	Papillary and follicular thyroid	c.470 T > C	51/602	(4,49%)	42/829	(2,53%)	1,8	[1,20 – 2,72]	0,004
Gąsior-Perczak et al. (14)	Polish	Papillary thyroid	c.470 T > C	189/1547	(12,3%)	25/468	(5,30%)	2,5	[2,47 – 3,79]	< 0,001
			c.1100delC	16/1547	(1,00%)	0/468	(0,00%)	NA	[NA]	NA
			c.444 + 1G > A	18/1547	(1,20%)	1/468	(0,20%)	7,1	[0,95 – 52,31]	0,056
			c.27417113-27422508del	10/1547	(0,60%)	2/468	(0,40%)	2,1	[0,48 -9,40]	0,319

Cybulski et al. published the first case-control study linking papillary thyroid cancer with an increased prevalence of germline *CHEK2* mutations. They found the prevalence of overall *CHEK2* mutations was 12,24% in patients with papillary thyroid cancer versus 5,53% in matched controls (no statistical analysis available). When considering the truncating mutations separately (IVS2 + 1G > A and 1100delC) the authors conclude there is a statistically significant association with thyroid cancer (OR 4,9; *p* = 0,0006) [7]. This association was corroborated in a larger case-control study by Siolek et al. The prevalence of four different *CHEK2* germline mutations was investigated in 468 patients with papillary thyroid cancer versus 468 age- and sex-matched controls. Patients with thyroid cancer had a significantly higher prevalence of truncating *CHEK2* mutations (IVS2 + 1G > A, c.1100delC or del 5395) than the control group (OR 5,7; *p* = 0,006) [8]. In the largest case-control study so far, Gasior-Perczak et al. found in the Polish population the *CHEK2* c.1100delC mutation to be present in 1% of papillary thyroid cancer patients (16/1547), and none in 468 healthy controls (no statistical analysis available). No statistically significant association was found between different individual truncating *CHEK2* variants and thyroid cancer. When pooling the truncating mutations however, there was a statistically significant association (OR 4,54; 95%CI [1,40 – 14,68]; *p* = 0,0116), which was driven primarily by the IVS2 + 1G > A variant [14]. A case-control study of patients with non-anaplastic thyroid cancer in an Iranian population found no germline *CHEK2* mutations in cases nor controls, though this probably reflects the low frequency of *CHEK2* mutations in the Middle Eastern population [15]. Two studies in the Polish population only investigated the I157T variant [16, 17].

These studies, together with the three other Polish studies mentioned previously, found an increased I157T carrier frequency in papillary thyroid cancer patients compared to controls with odds ratios between 1,9 to 3,0 [7, 8, 14, 16, 17].

### Prevalence of germline CHEK2 variants in thyroid cancer case series

In a Czech pediatric population of papillary thyroid cancer patients, 7/84 (8,4%) had a *CHEK2* variant. The majority carried the I157T (*n* = 5), with one c.1100delC carrier and 1 variant of uncertain significance (p.L467F) [18].

A conference abstract by Kamihara et al. reported genotyping 2678 thyroid cancer patients. In this ethnically more diverse population (66% Caucasian) *CHEK2* was the most frequently mutated gene (3,1%). About 90% of subjects were female, of which 50% had a history of breast cancer, implying an important selection bias. The specific *CHEK2* mutations were not reported, histological information was incomplete, and there was no control group [19])

## Discussion

### Is there a relation between risk of papillary thyroid cancer and germline CHEK2 mutations?

The cross-sectional study of Leedom et al. reports a statistically significant higher incidence of thyroid cancer history in carriers of truncating *CHEK2* variants versus non-carriers, but no conclusions can be drawn for papillary thyroid cancer specifically as no pathological details of thyroid cancer are available [11]. In the study by Näslund-Koch et al. there is a higher incidence of thyroid cancer in *CHEK2* c.1100delC carriers, though this difference is not reported as statistically significant [10]. These studies are all limited by their retrospective design, which has an inherent reporting bias, and are to be interpreted with caution. It should also be noted that Näslund-Koch only investigated c.1100delC whereas the two other studies investigated all variants. There seems to be no difference however in prevalence of thyroid cancer history between different *CHEK2* variants [4, 11].

Most of the case-control studies were conducted in the same, Polish, population. Here it was repeatedly shown that in patients with papillary thyroid carcinoma, the incidence of *CHEK2* truncating variants is significantly higher. There is possibly an elevated risk of thyroid cancers in CHEK2 c.444 + 1G > A carriers, although the number of carriers in these studies was limited. In other populations however these findings have thus far not been reproduced. Maybe a different environmental or genetic factor could be responsible for the stronger association between *CHEK2* variants and thyroid cancer in the Polish population. It has been proposed that differences in genetic background and exposure to environmental risk factors may modify specific risks, which implies that two individuals with the same mutation may face different cancer risks based on cultural background and genetic modifiers, specific to their population. For example, recently it has been reported that the increased risk of breast and prostate cancer associated with the *NBN* (657del5) founder mutation is modified by the presence of a common missense variant in the *NBN* gene (E185Q). The elevated cancer risk is limited to biallelic E185Q variant carriers (OR = 3.6; *p* < 0,001) and is not elevated in women with other E185Q genotypes (OR = 1.0; *p* = 0,9). On itself however *NBN* E185Q does not predispose to breast or prostate cancer [20].

### Should we screen carriers of a CHEK2 c.1100delC or other truncating variants for thyroid cancer?

Belgian national guidelines for *CHEK2* carriers recommend gynaecological follow-up from age 25 and breast cancer screening starting at age 35 in women, yearly PSA and digital prostate exam starting at age 50 in men, and colonoscopy every 5 years from age 40 (or 10y before youngest diagnosis of colorectal cancer in family) in both sexes. Current national and most international guidelines do not recommend screening for thyroid cancer in patients with *CHEK2* mutations [21–24]. Only the guidelines of the International Hereditary Cancer Centre propose screening for thyroid cancer in *CHEK2* carriers, based on the Polish case-control studies [25]. There is however insufficient evidence to support screening in other populations.

About 5% of non-medullary thyroid cancer present in familial form. Familial papillary carcinoma is considered when at least 3 first-degree relatives are diagnosed with papillary carcinoma, since a single first-degree relative with thyroid cancer may just be a sporadic event [22].. Current guidelines recommend yearly thyroid ultrasound screening in Cowden syndrome, Familial adenomatous polyposis (FAP), and the more rare Carney complex, Werner syndrome and DICER1 syndromes [22, 26]. In Cowden syndrome the lifetime risk of developing thyroid cancer is 6–35%, and annual screening is recommended [22, 26–28]. In FAP the absolute lifetime risk of thyroid cancer is much lower at 1–8%, nonetheless current guidelines recommend screening [22, 24]. However, screening in FAP is under debate considering low mortality due to thyroid cancer in this population (2/4820 patients; 0,04%) [29].

To justify a screening program a disease should be frequently occurring, screening should result in cancer detection at an earlier stage leading to a potentially curable treatment and benefits of early detection should outweigh the risks [29].

Current evidence suggests a low absolute risk of thyroid cancer in *CHEK2* mutation carriers, and these are mostly of differentiated, non-medullary histology. The available evidence likely overestimates the risk of developing thyroid cancer, because of selection and reporting bias. Furthermore, the findings in the Polish population have not been validated in other populations. There are no data available on thyroid specific mortality in *CHEK2* mutation carriers, nor is it clear if thyroid cancer in this population is more aggressive or presents at an earlier age. Thyroid carcinoma in general is associated with a high 5 year overall survival of 96, and > 90% of cases are diagnosed at an early, curable stage [30, 31]. Screening in comparable selected populations with low absolute prevalence results in a higher rate of papillary thyroid cancer detection and potentially harmful interventions, but no reduction in mortality [29].

## Conclusion

There is a low level of evidence for a slightly higher incidence of thyroid cancer in patients with (likely) pathogenic *CHEK2* mutations compared to non-carriers. Current guidelines do not recommend systematic screening. To evaluate whether systematic screening for thyroid cancer should be recommended in *CHEK2* carriers, a prospective cohort study, comparing the incidence of thyroid cancer in *CHEK2* mutation carriers and non-carriers should be set up, preferably including different ethnicities.

## Data Availability

All data analysed in this study are available in the manuscript.
